# A Preliminary Study of White Matter in Adolescent Depression: Relationships with Illness Severity, Anhedonia, and Irritability

**DOI:** 10.3389/fpsyt.2013.00152

**Published:** 2013-11-25

**Authors:** Sarah E. Henderson, Amy R. Johnson, Ana I. Vallejo, Lev Katz, Edmund Wong, Vilma Gabbay

**Affiliations:** ^1^Department of Psychiatry, Icahn School of Medicine at Mount Sinai, New York, NY, USA; ^2^Nathan S. Kline Institute for Psychiatric Research, Orangeburg, NY, USA

**Keywords:** depression, adolescent, white matter, diffusion tensor imaging, anhedonia, irritability

## Abstract

Major depressive disorder (MDD) during adolescence is a common and disabling psychiatric condition; yet, little is known about its neurobiological underpinning. Evidence indicates that MDD in adults involves alterations in white and gray matter; however, sparse research has focused on adolescent MDD. Similarly, little research has accounted for the wide variability of symptom severity among depressed teens. Here, we aimed to investigate white matter (WM) microstructure between 17 adolescents with MDD and 16 matched healthy controls (HC) using diffusion tensor imaging. We further assessed within the MDD group relationships between WM integrity and depression severity, as well as anhedonia and irritability – two core symptoms of adolescent MDD. As expected, adolescents with MDD manifested decreased WM integrity compared to HC in the anterior cingulum and anterior corona radiata. Within the MDD group, greater depression severity was correlated with reduced WM integrity in the genu of corpus callosum, anterior thalamic radiation, anterior cingulum, and sagittal stratum. However, anhedonia and irritability were associated with alterations in distinct WM tracts. Specifically, anhedonia was associated with disturbances in tracts related to reward processing, including the anterior limb of the internal capsule and projection fibers to the orbitofrontal cortex. Irritability was associated with decreased integrity in the sagittal stratum, anterior corona radiata, and tracts leading to prefrontal and temporal cortices. Overall, these preliminary findings provide further support for the hypotheses that there is a disconnect between prefrontal and limbic emotional regions in depression, and that specific clinical symptoms involve distinct alterations in WM tracts.

## Introduction

Major depressive disorder (MDD) in adolescence is a prevalent and disabling psychiatric illness associated with serious consequences including academic failure, social withdrawal, substance abuse, and most critically, suicide (1–5). Converging evidence derived from neuroimaging studies suggests that adolescent MDD entails morphological, functional, and neurochemical alterations (6–10). Importantly, since adolescence represents a critical period of rapid neuroplasticity [e.g., increased myelination, synaptic pruning; (11–13)], white matter (WM) alterations can contribute to the neurobiology of adolescent MDD. Indeed, in our prior investigation of gamma-Aminobutyric acid in the anterior cingulate cortex (ACC), we incidentally found reduced WM volume in adolescents with MDD compared to healthy controls [HC; (9)]. However, there has been sparse research focusing on WM alterations in this age group.

Diffusion tensor imaging (DTI) enables the non-invasive examination of *in vivo* structural connectivity by providing measures of WM microstructure and integrity based on the extent of water diffusion (14). Several DTI measures are typically quantified, with fractional anisotropy (FA) being the most commonly used to reflect WM integrity. Higher FA values suggest greater diffusion in the direction of the axon, and thus greater WM integrity. Other measures, including mean diffusivity (MD), radial diffusivity (RD), and axial diffusivity (AD), can also be determined to investigate different aspects of WM microstructure.

To date, most DTI research in MDD has investigated adults and consistently reported decreased FA in tracts connected to the prefrontal cortex (PFC) or tracts connecting the two hemispheres within the PFC (15, 16). Only one DTI study was carried out in adolescents with depression, demonstrating a similar pattern to adult MDD of reduced FA in tracts connected to the subgenual ACC and the PFC [i.e., uncinate fasciculus, inferior-fronto-occipital fasciculus, anterior cingulum, superior longitudinal fasciculus; (17)]. However, results may have been impacted by the concurrent use of psychotropic medication and past substance abuse in some subjects. Relatedly, medication-naïve adolescents with a familial risk for unipolar depression also demonstrated reduced FA compared to HC in similar tracts (18).

In this study, we aimed to expand on prior work by examining WM integrity in psychotropic medication-free adolescents with MDD compared to HC. Given the inherent heterogeneity of adolescent MDD, we further sought to identify specific WM alterations in relation to MDD severity as well as anhedonia and irritability – two core symptoms of adolescent MDD. Due to our desire to explore a range in depression severity, we included patients with mild to moderate severity. Based on others’ and our prior findings (8, 15–17), we hypothesized that adolescents with MDD would manifest less restricted diffusion (i.e., decreased WM integrity) compared to HC in tracts connecting frontal, striatal, and limbic regions. We also predicted that similar tracts would be associated with depression severity. For anhedonia, we expected reduced WM integrity in tracts that have been implicated in reward-related processing in the ventral striatum [i.e., subgenual cingulate, forceps minor, inferior-fronto-occipital fasciculus, anterior thalamic radiation (ATR), anterior limb of the internal capsule; (19)], and that these would be distinct from those associated with irritability. Finally, only one study has examined irritability in a clinical population [i.e., Huntington’s disease; (20)] and found involvement of the amygdala. As such, we predicted the impairment of tracts connecting to the amygdala (e.g., uncinate fasciculus, inferior-fronto-occipital fasciculus, inferior longitudinal fasciculus).

## Materials and Methods

### Participants

The sample population partially overlapped with that used for our previously published resting state functional magnetic resonance imaging research (8), and was comprised of 17 adolescents with MDD (ages 13–20 years, *M* = 16.8, *SD* = 2.2, 8 female) and 16 HC (ages 13–19 years, *M* = 16.4, *SD* = 1.4, 10 female) group matched for age, and all were right-handed. Depressed adolescents were recruited through the New York University (NYU) Child Study Center, the Bellevue Hospital Center Department of Psychiatry, and local advertisements in the NY metropolitan area. HC were recruited from the greater NY metropolitan area through local advertisements and from the families of NYU staff. The study was approved by the NYU School of Medicine and the Icahn School of Medicine at Mount Sinai institutional review boards. Prior to enrollment, study procedures were explained to the subjects and parents. Written informed consent was provided by participants age 18 and older; those under age 18 provided signed assent and a parent/guardian provided signed informed consent.

#### Inclusion and exclusion criteria

All subjects were ≤20 years old and did not present with any significant medical or neurological disorders. Other exclusionary criteria consisted of an IQ < 80, MRI contraindications as assessed by a standard screening form, a positive urine toxicology test or a positive urine pregnancy test in females.

All MDD subjects met the DSM-IV-TR diagnosis of MDD with a current episode ≥8 weeks duration, and raw severity score ≥40 (i.e., T score ≥ 63) during the initial evaluation on the Children’s Depression Rating Scale-Revised (CDRS-R), and were psychotropic medication-free for at least 7 half-lives of the medication. To explore a wider range of depression severity we included patients presenting with mild to severe depression on the date of the scan. Exclusionary criteria for the MDD group included current or past DSM-IV-TR diagnoses of bipolar disorder, schizophrenia, pervasive developmental disorder, panic disorder, obsessive-compulsive disorder, conduct disorder, Tourette’s disorder, or a substance-related disorder in the past 12 months. Current diagnoses of post-traumatic stress disorder or an eating disorder were also exclusionary. In addition, acute suicidality requiring immediate inpatient admission was exclusionary. HC subjects did not meet the criteria for any major current or past DSM-IV-TR diagnoses and had never received psychotropic medication.

### Clinical assessments

All subjects were assessed by a board-certified child and adolescent psychiatrist or a clinical psychologist at the NYU Child Study Center. Clinical diagnoses were established using the Schedule for Affective Disorders and Schizophrenia for School-Age Children-Present and Lifetime Version [KSADS-PL; (21)], a semi-structured interview performed with both the subjects and their parents. Depression severity was assessed by the CDRS-R and the Beck Depression Inventory, second edition [BDI-II; (22)]. Additionally, suicidality and anxiety were assessed using the Beck Scale for Suicidal Ideation [BSSI; (23)] and the Multidimensional Anxiety Scale for Children [MASC; (24)], respectively. The Kaufman Brief Intelligence Test (25) or the Wechsler Abbreviated Scale of Intelligence (26) were used to estimate IQ. Urine toxicology and pregnancy tests were administered on the day of the scan.

#### Anhedonia

Our approach to quantifying anhedonia allows for clinician- and self-rated assessments to contribute equally to the anhedonia score (range 1–13). As in our previous studies (8, 9, 27), the score for each subject was computed by summing the responses to three items associated with anhedonia from the clinician-rated CDRS-R (item 2: “difficulty having fun;” scale of 1–7) and the self-rated BDI-II (items 4: “loss of pleasure” and 12: “loss of interest;” scales of 0–3).

#### Irritability

Our approach again combined both clinician- and self-rated assessments to contribute to the irritability score (range 1–10). The score for each subject was computed by summing the responses to the items associated with irritability from the CDRS-R (item 8: “irritability;” scale of 1–7) and the BDI-II (item 17: “irritability;” scale of 0–3).

### Data acquisition and analysis

Diffusion data were acquired on a Siemens Allegra 3.0 T scanner at the NYU Center for Brain Imaging using a single-channel head coil. Diffusion-weighted echo-planar images (EPI) were acquired along 12 diffusion gradient directions for acquisition of 35 slices through the whole brain (TR = 6000 ms, TE = 82 ms, flip angle = 90°, *b* value = 1000 s/mm^2^, FOV = 192 mm, 128 × 128 matrix, slice thickness = 2.5 mm, with four averages). High-resolution T1-weighted anatomical images were acquired using a magnetization-prepared gradient echo sequence (TR = 2530 ms; TE = 3.25 ms; TI = 1100 ms; flip angle = 7°; 128 slices; FOV = 256 mm; acquisition voxel size = 1.3 mm × 1 mm **× **1.3 mm).

All preprocessing was performed using FMRIB’s Software Library (FSL; Oxford, UK): FMRIB’s Diffusion Toolbox (FDT). Preprocessing began with eddy current correction to correct for gradient-coil distortions and small head motions, using affine registration to a b0 reference volume. A diffusion tensor model was fitted to each voxel along the principal λ_1_, λ_2_, λ_3_ directions to generate FA, MD, RD, and AD. We then implemented FSL’s tract-based spatial statistics pipeline [TBSS; (28)], in which a non-linear registration aligned each subject to the FMRIB58_FA template in 1 mm × 1 mm × 1 mm standard space, and then warped each subject into standard Montreal Neurological Institute space (MNI152). A WM “skeleton” was then generated representing a single line running down the centers of all of the common WM fibers by using an FA cut-off of 0.2, and relevant diffusivity measures (i.e., FA, MD, RD, AD) were projected onto the skeleton. Group statistical analysis was then conducted only on voxels within the WM skeleton mask, therefore restricting the voxel-wise analysis only to voxels with high confidence of lying within equivalent major WM pathways in each individual.

In order to assess differences in FA, MD, RD, and AD between the MDD and HC groups, we used FSL’s Randomise with 5000 permutations to perform voxel-wise independent samples *t*-tests using voxel-based thresholding while controlling for age and sex. Group comparisons did not withstand stringent correction for multiple comparisons using family-wise error correction (FWE; implemented by Randomise) or FDR correction (implemented by FSL’s FDR program). As such, we performed a second, more exploratory analysis in which we accepted clusters of at least 10 contiguous voxels at *p* < 0.001.

To investigate relationships with depression severity, anhedonia, and irritability, Randomise was again used to perform a series of one-sample *t*-tests using the score for each category, respectively, while controlling for age and gender. Due to the low variability in scores in the HC group, as well as the nature of the study topic, the analysis was limited to the MDD group whose scores were normally distributed. Once again, tests did not withstand correction for multiple comparisons and we used the more exploratory approach of accepting clusters of at least 10 contiguous voxels at *p* < 0.001. We used FSL’s cluster program to extract all clusters across the brain, and anatomical localization of each cluster was determined using the FSLView atlas toolbox and the relevant gray matter (Harvard-Oxford Cortical/Subcortical) and WM (Johns Hopkins University WM tractography) atlases. Brain imaging results were prepared for display using FSL’s tbss_fill script, which displays results superimposed upon the WM skeleton from the group TBSS analysis.

## Results

### Participants

Demographic and clinical characteristics are summarized in Table 1. One subject with MDD had been treated with Lexapro and Ambien for 7 months, but was medication-free for approximately 14 months prior to scanning. A second subject with MDD had a brief trial with Prozac which was self-discontinued prior to participation in this study. All other subjects were psychotropic medication-naïve. Fifteen subjects with MDD had experienced only one episode of depression, with length of episode ranging from 4 to 48 months, and two patients reported having two distinct episodes. Two Shapiro–Wilk tests revealed that anhedonia and irritability were both normally distributed within the MDD group, *p*s = 0.81, 0.48, respectively. Depression severity (excluding the anhedonia- or irritability-related items) was significantly correlated with both severity of anhedonia (*r* = 0.66, *p* < 0.005) and irritability (*r* = 0.66, *p* < 0.005) within our MDD sample. Anhedonia and irritability were not correlated (*r* = 0.37, *p* = 0.15).

**Table 1:** **Demographic and clinical characteristics of adolescents with major depressive disorder (MDD) and healthy controls**.

Characteristic	MDD subjects (*N* = 17)	Healthy controls (*N* = 16)
Age (range)	16.8 ± 2.2 (13–20)	16.4 ± 1.4 (13–19)
Gender (female/male)	8/9 (47/53%)	10/6 (63/38%)^a^
Ethnicity (Caucasian/African American/Hispanic/Asian/other)	9/2/5/0/1 (53/12/29/0/6%)	6/5/1/1/3 (38/31/6/6/19%)
**ILLNESS HISTORY**		
Current episode duration in months (range)	24.4 ± 16.0 (7–72)	0
Number of MDD episodes	1 (*n* = 15), 2 (*n* = 2)	0 (*n* = 16)
History of suicide attempts (range)	0.2 ± 0.5 (0–2)	0
Medication-naïve/medication-free	15/2 (88/12%)	16/0 (100/0%)
CDRS-R (range)^b^	45.7 ± 9.7 (29–64)	19.4 ± 2.7 (17–27)
Anhedonia (range)	5.76 ± 2.28 (2–10)	1.44 ± 0.73 (1–3)
Irritability (range)	4.71 ± 1.40 (2–7)	1.18 ± 0.83 (0–3)
BDI-II (range)^c^	19.2 ± 10.4 (1–37)	1.9 ± 2.6 (0–10)
BSSI (range)^d^	2.0 ± 4.2 (0–14)	0.1 ± 0.3 (0–1)
MASC (range)^e^	44.2 ± 21.3 (11–75)	33.5 ± 12.4 (9–55)
**CURRENT COMORBIDITY**		
ADHD^f^	1 (6%)	0
Any anxiety disorder	8 (47%)	0
GAD^g^	7 (41%)	0

*^a^ Respective percentages (may not add up to 100% due to rounding)*.

*^b^ Children’s depression rating scale – revised*.

*^c^ Beck depression inventory, 2nd ed*.

*^d^ Beck scale for suicidal ideation*.

*^e^ Multidimensional anxiety scale for children*.

*^f^ Attention deficit hyperactivity disorder*.

*^g^ Generalized anxiety disorder*.

### Whole-brain group comparison

**Figure 1:**
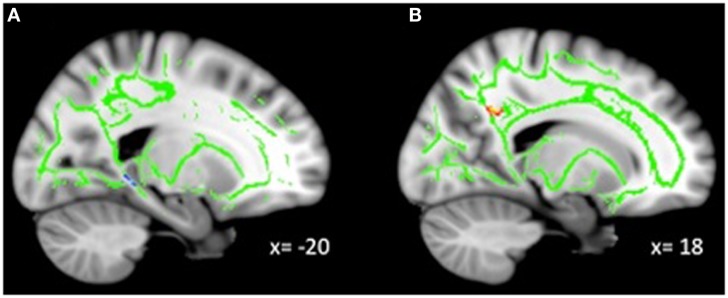
**(A)** Increased WM integrity in the MDD group vs. HC in the posterior cingulum near the hippocampus; **(B)** decreased WM integrity in the MDD group vs. HC in the anterior cingulum near the precuneus.

No voxel-wise group comparisons for FA, MD, RD, or AD withstood correction for multiple comparisons. An exploratory analysis using a threshold of *p* < 0.001, uncorrected with clusters exceeding 10 contiguous voxels, revealed 4 significant clusters (Table 2, Figure 1). Compared with HC, the MDD group had lower FA in the anterior cingulum, and lower AD in the anterior corona radiata (ACR). However, the MDD group also had greater FA and lower RD in the posterior cingulum compared to HC.

**Table 2:** **Voxel-wise group comparison results**.

Region	Tract	COG coordinates			Cluster size	Values mean (SD) HC/MDD
		*x*	*y*	*z*	
**FA: MDD > HC**						
L cerebral WM (hippocampus)	Posterior cingulum	−20	−39	−5	16	0.45 (0.06)
						0.57 (0.08)
**FA: HC > MDD**						
R cerebral WM (precuneus)	Anterior cingulum	18	−51	34	10	0.50 (0.05)
						0.43 (0.04)
**MD: MDD > HC**						
None
**MD: HC > MDD**						
None
**RD: MDD > HC**						
None
**RD: HC > MDD**						
L cerebral WM (hippocampus)	Posterior cingulum	−20	−39	−5	16	0.0006 (7E−05)
						0.0005 (8E−05)
**AD: MDD > HC**						
None
**AD: HC > MDD**						
L cerebral WM (putamen)	ACR	−23	22	−5	10	0.0013 (9E−05)
						0.0012 (8E−05)

*Units for AD and RD = mm^2^/s. Coordinates in MNI space. Threshold *p* < 0.001, uncorrected, *k* > 10. COG, center of gravity; FA, fractional anisotropy; MD, mean diffusivity; RD, radial diffusivity; AD, axial diffusivity; WM, white matter; L, left; R, right; ACR, anterior corona radiata*.

### Whole-brain correlations with depression severity in MDD

**Figure 2:**
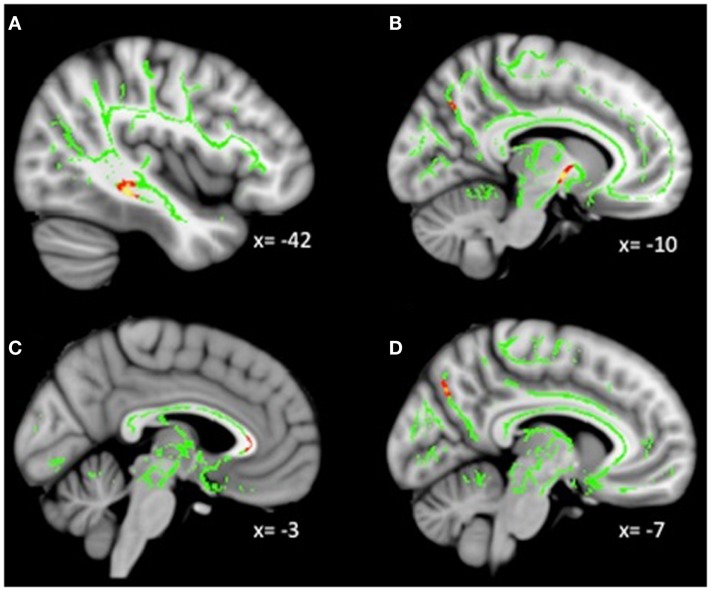
**Decreased WM integrity as depression severity increased in the (A) sagittal stratum near the PHG; (B) ATR near the pallidum; (C) genu of the corpus callosum and anterior cingulate; (D) anterior cingulate near the precuneus**. Key: PHG, parahippocampal gyrus; ATR, anterior thalamic radiation.

Exploratory analyses revealed a total of 16 uncorrected clusters, with 5 overlapping clusters between the 4 diffusivity measures (Table 3, Figure 2). As depression severity increased, FA decreased in the genu of the corpus callosum, the sagittal stratum, the ATR, and the anterior cingulum. Additionally, a positive correlation between MD and depression severity was found in the same sagittal stratum cluster as the FA analysis, as well as in clusters in the ATR and corticospinal tract. Similarly, illness severity was positively correlated with RD in the same sagittal stratum cluster as the FA and MD analyses, the same genu of the corpus callosum and ATR clusters as the FA analysis, the same ATR cluster as the MD analysis, and a cluster in the superior longitudinal fasciculus (SLF). Finally, increased illness severity was associated with increased AD in the same corticospinal cluster as the MD analysis as well as in clusters in the inferior-fronto-occipital fasciculus (IFOF), the SLF, and fibers projecting to the orbitofrontal cortex (OFC).

**Table 3:** **Voxel-wise correlations with depression severity (CDRS-R)**.

Region	Tract	COG			Cluster size
		*x*	*y*	*z*	
**FA: POSITIVE RELATIONSHIP**					
None
**FA: NEGATIVE RELATIONSHIP**					
L cerebral WM (PHG)	Sagittal stratum (ILF + IFOF)	−42	−35	−9	29
L cerebral WM (pallidum)	ATR	−10	−3	−3	20
L cerebral WM (ACC)	Genu of corpus callosum	−2	30	4	14
L cerebral WM (precuneus)	Anterior cingulum	−7	−72	39	11
**MD: POSITIVE RELATIONSHIP**					
L cerebral WM (PHG)	Sagittal stratum (ILF + IFOF)	−41	−33	−13	20
R cerebral WM (pallidum)	ATR	14	−3	3	11
R cerebral WM (postcentral gyrus)	Corticospinal	22	−40	47	10
**MD: NEGATIVE RELATIONSHIP**					
None
**RD: POSITIVE RELATIONSHIP**					
L cerebral WM (PHG)	Sagittal stratum (ILF + IFOF)	−42	−35	−10	45
L cerebral WM (pallidum)	ATR	−10	−3	−4	17
R cerebral WM (supramarginal gyrus)	SLF	30	−39	37	13
L cerebral WM (ACC)	Genu of corpus callosum	−2	29	4	14
R cerebral WM (pallidum)	ATR	14	−2	3	10
**RD: NEGATIVE RELATIONSHIP**					
None
**AD: POSITIVE RELATIONSHIP**					
R cerebral WM (postcentral gyrus)	Corticospinal	22	−33	44	25
R cerebral WM (precuneus)	IFOF	23	−55	30	17
L cerebral WM (MFG)	SLF	−31	21	25	10
R cerebral WM (OFC)	Frontal projection fibers	−24	19	−18	10
**AD: NEGATIVE RELATIONSHIP**					
None

*Coordinates in MNI space. Threshold *p* < 0.001, uncorrected, *k* > 10. COG, center of gravity; L, left; R, right; FA, fractional anisotropy; MD, mean diffusivity; RD, radial diffusivity; AD, axial diffusivity; WM, white matter; PHG, parahippocampal gyrus; ACC, anterior cingulate cortex; MFG, medial frontal gyrus; OFC, orbitofrontal gyrus; ILF; inferior longitudinal fasciculus; IFOF, inferior-fronto-occipital fasciculus; ATR, anterior thalamic radiation; SLF, superior longitudinal fasciculus*.

### Whole-Brain correlations with anhedonia in MDD

**Figure 3:**
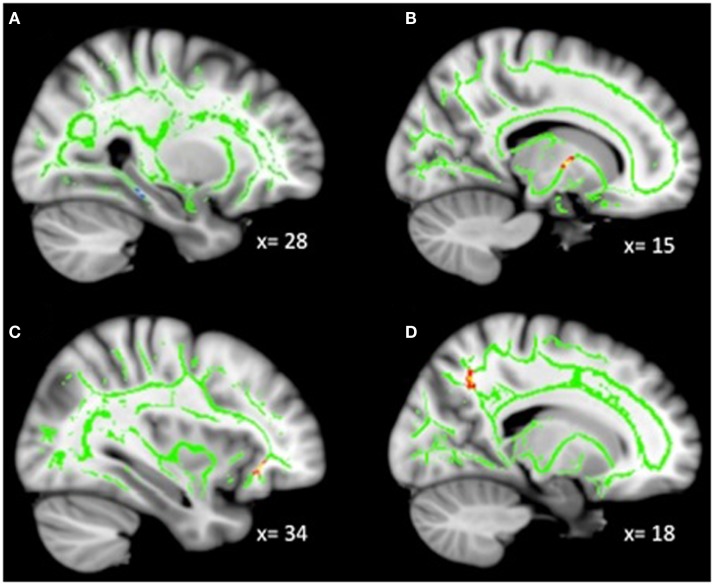
**(A)** Increased WM integrity as anhedonia increased in the posterior cingulum near the hippocampus; decreased WM integrity as anhedonia increased in the **(B)** anterior limb of the internal capsule; **(C)** IFOF near the OFC; **(D)** posterior cingulum near the precuneus. Key: IFOF, inferior-fronto-occipital fasciculus; OFC, orbitofrontal cortex.

Analyses revealed a total of 14 uncorrected clusters, with 2 overlapping clusters between FA and RD (Table 4, Figure 3). As anhedonia increased, FA increased in a cluster in the posterior cingulum near the hippocampus – similar to a cluster from the group comparison analysis – and decreased in the anterior limb of the internal capsule, OFC projection fibers, and the posterior cingulum near the precuneus. Furthermore, anhedonia was positively correlated with MD in OFC projection fibers, the external capsule, and the sagittal stratum. Additionally, increased anhedonia severity was associated with greater RD in the same posterior limb of the internal capsule and posterior cingulum clusters as the FA analysis, the same OFC projection fibers as the MD analysis, and clusters in the ATR and corticospinal tract. Finally, there were positive correlations between anhedonia and AD in the corticospinal tract and projection fibers into the occipital cortex.

**Table 4:** **Voxel-wise correlations with anhedonia**.

Region	Tract	COG			Cluster size
		*x*	*y*	*z*	
**FA: POSITIVE RELATIONSHIP**					
R cerebral WM (hippocampus)	Posterior cingulum	25	−31	−14	13
**FA: NEGATIVE RELATIONSHIP**					
R cerebral WM (thalamus)	Anterior limb IC (ATR)	14	−2	4	14
R cerebral WM (OFC)	IFOF	34	31	−6	12
L cerebral WM (precuneus)	Posterior cingulum	18	−58	42	10
**MD: POSITIVE RELATIONSHIP**					
R cerebral WM (OFC)	Projection fibers	17	20	−18	14
L cerebral WM (putamen)	External capsule (IFOF)	−32	−22	2	11
L cerebral WM (fusiform gyrus)	Sagittal stratum (ILF + IFOF)	−40	−35	−14	11
**MD: NEGATIVE RELATIONSHIP**					
None
**RD: POSITIVE RELATIONSHIP**					
L cerebral WM (pallidum)	ATR	−10	−2	−3	24
R cerebral WM (OFC)	Projection fibers	17	20	−17	14
R cerebral WM (precuneus)	IFOF	18	−58	44	11
R cerebral WM (postcentral gyrus)	Corticospinal	18	−39	57	10
R cerebral WM (thalamus)	Anterior limb IC (ATR)	14	−2	4	10
**RD: NEGATIVE RELATIONSHIP**					
None
**AD: POSITIVE RELATIONSHIP**					
R cerebral WM (postcentral gyrus)	Corticospinal	22	−37	46	34
L cerebral WM (occipital)	Projection fibers	−15	−65	50	12
**AD: NEGATIVE RELATIONSHIP**					
None

*Coordinates in MNI space. Threshold *p* < 0.001, uncorrected, *k* > 10. COG, center of gravity; L, left; R, right; FA, fractional anisotropy; MD, mean diffusivity; RD, radial diffusivity; AD, axial diffusivity; WM, white matter; OFC, orbitofrontal gyrus; IC, internal capsule; ATR, anterior thalamic radiation; IFOF, inferior-fronto-occipital fasciculus; ILF, inferior longitudinal fasciculus*.

### Whole-brain correlations with irritability in MDD

**Figure 4:**
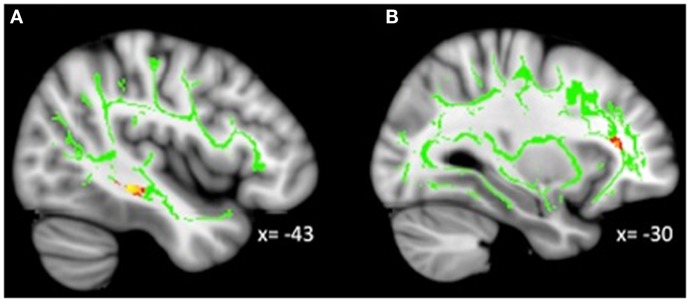
**Decreased WM integrity as irritability increased in the (A) sagittal stratum in the IT; (B) IFOF near the MFG**. Key: IT, inferior temporal cortex; IFOF, inferior-fronto-occipital fasciculus; MFG, medial frontal gyrus.

Analyses revealed a total of 14 uncorrected clusters, with 2 overlapping clusters (Table 5, Figure 4). As irritability increased, FA decreased in clusters in the sagittal stratum and IFOF, while MD increased in the same sagittal stratum cluster as well as in clusters in the ACR, SLF, and IFOF. For RD, positive correlations with irritability were evident in the same sagittal stratum cluster, the anterior limb of the internal capsule, and the SLF. Positive correlations were also found between AD and irritability in the same ACR cluster as the MD analysis as well as in the IFOF, the corticospinal tract, and the SLF.

**Table 5:** **Voxel-wise correlations with irritability**.

Region	Tract	COG			Cluster size
		*x*	*y*	*z*	
**FA: POSITIVE RELATIONSHIP**					
None					
**FA: NEGATIVE RELATIONSHIP**					
L cerebral WM (IT)	Sagittal stratum (ILF + IFOF)	−43	−33	−9	51
L cerebral WM (MFG)	IFOF	−31	33	12	13
**MD: POSITIVE RELATIONSHIP**					
L cerebral WM (IT)	Sagittal stratum (ILF + IFOF)	−42	−34	−9	82
L cerebral WM (putamen)	ACR (ATR)	−22	17	17	19
L cerebral WM (IT)	SLF	−47	−39	−4	13
L cerebral WM (lingual gyrus)	IFOF	−14	−86	−6	11
**MD: NEGATIVE RELATIONSHIP**					
None					
**RD: POSITIVE RELATIONSHIP**					
L cerebral WM (IT)	Sagittal stratum (ILF + IFOF)	−45	−34	−8	99
R cerebral WM (supramarginal gyrus)	SLF	34	−43	33	15
R cerebral WM (supramarginal gyrus)	SLF	38	−43	26	12
R cerebral WM (putamen)	Anterior limb IC (ATR)	14	7	6	12
**RD: NEGATIVE RELATIONSHIP**					
None
**AD: POSITIVE RELATIONSHIP**					
R cerebral WM (postcentral gyrus)	Corticospinal	22	−30	46	22
R cerebral WM (precuneus)	IFOF	23	−55	32	18
L cerebral WM (MT)	SLF	−49	−46	−1	16
L cerebral WM (putamen)	ACR	−22	18	18	10
**AD: NEGATIVE RELATIONSHIP**					
None

*Coordinates in MNI space. Threshold *p* < 0.001, uncorrected, *k* > 10. COG, center of gravity; L, left; R, right; FA, fractional anisotropy; MD, mean diffusivity; RD, radial diffusivity; AD, axial diffusivity; WM, white matter; IT, inferior temporal gyrus; MFG, medial frontal gyrus; MT, middle temporal gyrus; ILF, inferior longitudinal fasciculus; IFOF, inferior-fronto-occipital fasciculus; ACR, anterior corona radiata; ATR, anterior thalamic radiation; SLF, superior longitudinal fasciculus; IC, internal capsule*.

## Discussion

Consistent with our hypotheses, analyses revealed reduced WM integrity (i.e., decreased FA, and increased MD, RD, AD) in the MDD group compared to HC as well as in the more severely depressed, anhedonic, and irritable patients. Furthermore, despite significant correlations between the two dimensional measures and depression severity, we found distinct WM alterations for both anhedonia and irritability that differed from those for depression severity. Reduced integrity was found in fronto-striatal and thalamic tracts, the corpus callosum, and tracts connected to the inferior temporal (IT) cortex. Additionally, reduced integrity in the sagittal stratum was consistently found in our analyses to be correlated with increasing depression severity, anhedonia, and irritability. Unexpectedly, analyses also revealed a cluster in the posterior cingulum near the hippocampus which demonstrated more anisotropic diffusion, both as anhedonia increased and in the MDD group vs. HC. Finally, it is interesting to note that in many of our analyses there was overlap in the clusters demonstrating a relationship with FA and RD, potentially suggesting that structural issues related to RD are driving the observed relationships with FA in this and other studies.

### Group differences in WM integrity

Group differences were observed in both the posterior and anterior cingulum. Specifically, depressed adolescents demonstrated decreased WM integrity in the anterior cingulum near the precuneus, and increased integrity in the posterior cingulum near the hippocampus, compared to HC. The cingulum connects the cingulate and entorhinal cortices and is broadly involved in attention, memory, and emotions (29, 30). The anterior portion of the cingulate has been implicated in emotional processing and depression (31). Altered functioning, connectivity, and diffusion around the precuneus are frequently reported in MDD (32, 33). Given the role of the precuneus in self-related processes, and that self-processing is typically altered in depression (34), this potentially suggests that reduced WM integrity contributes to altered functioning in this region early in the course of the disease.

The MDD group also demonstrated more coherent diffusion in the posterior cingulum near the hippocampus. The posterior cingulate is involved in cognitive functions including attention and memory (35). Functional hyperactivity in the hippocampus (36–38), as well as decreased hippocampal volume (39), are consistent findings in adult MDD. Given the role of the hippocampus in learning and memory (40), but also in the regulation of motivation and emotion (41, 42), this region is critical to carrying out normal behaviors that may be altered in depression. Furthermore, greater WM integrity in tracts leading to the hippocampus would be consistent with the literature demonstrating hyperactivity of this region in non-medicated MDD patients. Overall, the categorical comparison between depressed adolescents and HC revealed differences in an important tract connecting prefrontal and limbic regions.

### Depression severity and WM integrity

Our use of an approach that accounts for a range of depression severity in our sample revealed a pattern of reduced WM integrity as depression severity increased. Specifically, we found reduced integrity in the genu of the corpus callosum, a region that connects prefrontal and orbitofrontal cortices. Many studies have documented altered diffusivity in the genu (16) as well as reduced volume (6, 43–45). Given that the prefrontal and orbitofrontal cortices are involved in critical processes, including decision-making, attention, reward processing, and the evaluation and regulation of emotion (46–48), an interruption in communication between these areas has implications for depression and mood disorders.

Additionally, we found decreased integrity in the sagittal stratum with increasing severity, not only in this analysis, but also in the dimensional analyses within the MDD population. The sagittal stratum is a complex fiber bundle connecting the occipital cortex to the rest of the brain, and includes fibers from many major tracts including the ILF and IFOF (49). The ILF and IFOF both connect the occipital cortex to temporal limbic structures (i.e., amygdala, hippocampus) and the PFC, although the IFOF connects directly to the OFC and the ILF does so indirectly through the uncinate fasciculus (50). Therefore, both tracts are involved in connecting visual information with areas involved in emotional memories, judgments, and behaviors. A meta-analysis of diffusion studies of patients with MDD found WM alterations in both the ILF and IFOF (16). Additionally, alterations in WM have been found in the IFOF for depressed adolescents (17), adolescents with a familial risk for depression (18), and adults with MDD (51).

We also observed reduced integrity with increasing severity in bilateral clusters in the ATR near the pallidum. The ATR connects thalamic nuclei with the PFC through the anterior limb of the internal capsule. Reduced WM integrity has been reported in the ATR in several studies of depressed adults (16). Furthermore, given the role of the thalamus in motivation and goal pursuit (52, 53), altered connectivity within this circuit could contribute to the motivational deficits associated with depression.

Additionally, increased illness severity was associated with reduced integrity in the corticospinal tract near the postcentral gyrus. The corticospinal tract transmits motor impulses from the motor and premotor cortices to the spinal cord. Although this was an unexpected finding, motor disturbances and retardation are a relevant clinical symptom of depression (54). In this way, altered diffusivity may be related to the observed slowing and impairment of motor functions. Finally, we again found decreased integrity with increasing severity in the previously described anterior cingulum near the precuneus, which is consistent with the findings from our group analysis. Overall, our analysis with varied levels of depression severity was more robust than the categorical comparison and revealed a more extensive network of reduced WM integrity.

### Anhedonia and WM integrity

The dimensional analysis with anhedonia revealed an association between increased anhedonia and reduced integrity in the anterior limb of the internal capsule near the thalamus, a tract implicated in reward processing (19). The anterior limb of the internal capsule connects the thalamus with cingulate and prefrontal cortices, which are heavily involved in motivation, decision-making, and evaluating the saliency of emotional and rewarding stimuli. Additionally, increased anhedonia was correlated with reduced integrity in tracts (i.e., IFOF, projection fibers) connected to the posterior lateral OFC (BA 47), an area involved in many functions including emotional and reward processing, complex learning, and the inhibition of responses (46, 47, 55). Depressed patients have demonstrated reduced gray matter volume in the posterior lateral OFC as well as altered functional responses to emotional stimuli, reward processing, and reversal learning (56).

We also found reduced integrity in the external capsule as anhedonia increased. The external capsule contains cholinergic fibers projecting from the basal forebrain to the cerebral cortex. Reduced integrity in the external capsule has been found previously in adult MDD (57, 58). Furthermore, we once again found reduced integrity in the previously discussed sagittal stratum and posterior cingulum near the precuneus with greater symptom severity. Finally, the analysis revealed increased integrity with increased anhedonia in the posterior cingulum near the hippocampus, in an area fairly close to the cluster that showed increased integrity in the MDD group in our categorical comparison. In this way, it is possible that anhedonic symptoms are related to the group differences we observed. Given the previously discussed role of the hippocampus and limbic system in the regulation of motivation and emotion, the relationship between hippocampal functioning and anhedonia represents an important area for future research.

### Irritability and WM integrity

As predicted, we found decreased integrity as irritability increased in a tract near the amygdala (i.e., sagittal stratum including the ILF and IFOF). However, increased irritability was correlated with decreased integrity in tracts primarily connecting to prefrontal and occipital cortices. We also found clusters in the previously discussed IFOF, although one was in the lingual gyrus while the other was in the middle frontal gyrus. The lingual gyrus has been implicated in processing emotional faces (59), which is typically altered in MDD (60). Altered cerebral blood flow and resting state connectivity have been demonstrated in the lingual gyrus in adults with MDD (61, 62). Additionally, decreased integrity in WM has previously been found around the middle frontal gyrus (63), an area broadly involved in a variety of higher-level cognitive processes (64) which are often compromised in MDD.

Reduced integrity related to elevated irritability was also found in the ACR, which connects the striatum to the ACC. Reduced integrity has previously been demonstrated in the ACR in pediatric bipolar patients (65), and dysfunctional activity in the ACC is typically considered a hallmark of depression (37, 42, 66–70). Furthermore, altered intrinsic functional connectivity (i.e., resting state) between the striatum and ACC has been documented for depressed adolescents (8). Finally, a cluster in the SLF in the IT gyrus was found. The SLF is a major bidirectional association tract connecting large parts of the frontal cortex with the parietal, temporal, and occipital lobes. Less restricted diffusion in the SLF has been previously demonstrated for depressed adolescents (17), adolescents with a genetic risk for depression (18), and adults with MDD (71).

### Measures of WM integrity

Although a complete discussion of RD and AD goes beyond the scope of this paper, it is interesting to note that for both the categorical and dimensional analyses we found overlap in clusters with reduced FA and increased RD, but no overlapping relationships with AD. Increased RD may be caused by disturbances in myelin, whereas decreased AD has been suggested to reflect disrupted axonal integrity (72–74). As such, our findings and those from previous research may reflect that alterations in FA for MDD are being driven more by issues of myelination than axonal integrity. However, further research is needed to replicate and expand upon a possible mechanism.

### Limitations and future directions

Although our findings are consistent with other clinical studies investigating altered WM in depressed adolescents, it should be noted that very liberal thresholds were used for the analyses and the inability to correct for multiple comparisons is an issue of concern. Although our statistical methodology and sample size were comparable to those of other studies of clinical populations using DTI (17, 63, 75), it is possible that the sample sizes used in many clinical studies are not large enough to produce adequate statistical power. In this way, it is difficult to adequately balance the concerns of committing a Type I error by not correcting while also avoiding a Type II error due to small sample sizes and reduced statistical power. Therefore, our findings are considered preliminary. Furthermore, the inclusion of patients with milder symptomatology may have weakened our ability to detect group differences.

Although small sample sizes may be a possible explanation for the relatively weak results in our and other clinical studies of adolescent depression, another possibility is that the adolescent brain is still malleable and the alterations in WM structure may not fully take hold until adulthood (11). Therefore, it is even more pressing to understand a neuroimmunological model of depression and the factors that may contribute to changes in WM before chronicity begins to take effect. For example, given past findings that depressed adolescents exhibit higher levels of circulating inflammatory cytokines (76), one possible explanation for the observed reduction in FA in adult MDD is that it may reflect effects of chronic low grade inflammation. Additionally, given our previous research on fronto-striatal functional connectivity in MDD, future studies should investigate altered WM microstructure using a targeted tractography approach. Finally, further research is needed to investigate this hypothesis and other models of the systemic consequences of depression. To this end, a better understanding of what FA, MD, AD, and RD illustrate in an adolescent population, as well as the factors that contribute to these diffusivity measures, is needed in the field.

## Conclusion

Our investigation of altered WM microstructure in medication-free adolescents with MDD revealed a general pattern of impaired WM integrity in the depressed adolescents, and as depression severity, anhedonia, and irritability increased. Our findings are consistent with an overall hypothesis that depression, even in adolescence, involves a disconnection of prefrontal, striatal, and limbic emotional areas (16). Although this represents a good step toward understanding depression during this critical period, more research is needed to understand the factors that ultimately contribute to altered WM microstructure in order to develop potential interventions.

## Conflict of Interest Statement

The authors declare that the research was conducted in the absence of any commercial or financial relationships that could be construed as a potential conflict of interest.
